# Regulatory Effect of *Lactobacillus brevis* Bmb6 on Gut Barrier Functions in Experimental Colitis

**DOI:** 10.3390/foods9070864

**Published:** 2020-07-02

**Authors:** Mi-Young Shin, Cheng-Chung Yong, Sejong Oh

**Affiliations:** 1 Microbiology and Functionality Research Group, World Institute of Kimchi, Gwangju 61755, Korea; smy676@wikim.re.kr; 2 Division of Animal Science, Chonnam National University, Gwangju 61186, Korea; yongchengchung@gmail.com

**Keywords:** *Lactobacillus*, colitis, gut barrier function, tight junction protein

## Abstract

The integrity of gut barrier functions is closely associated with the pathogenesis of colitis. It is speculated that *Lactobacillus brevis* Bmb6 alleviates colitis by improving the tight junction (TJ) of the inflamed intestinal epithelial layer. In the present study, the regulatory effects of *L. brevis* Bmb6 on the TJ barrier to ameliorate colitis-symptoms were investigated. Preliminary screening showed that *L. brevis* Bmb6 exhibited strong acid and bile acid tolerance, along with antioxidants and β-galactosidase activities. In a 14-day dextran sulfate sodium (DSS)-induced colitis mouse model, treatment with *L. brevis* Bmb6 significantly decreased in the disease activity index score. In addition, histological analyses showed that treatment with *L. brevis* Bmb6 protected the structural integrity of the intestinal epithelial layer and mucin-secreting goblet cells from DSS-induced damage, with only slight infiltration of immune cells. Interestingly, western blotting analyses showed that the expression of the TJ protein, zona occluden-1, was restored in Bmb6-treated mice, but not in DSS-induced mice. Consistently, the gene expression of inflammatory cytokines (tumor necrosis factor-α and interferon-γ) was also suppressed in the Bmb6-treated mice. Hence, our findings suggest that suppression of inflammatory conditions enhanced expression of TJ protein, ZO-1, or vice versa, contributing to a colitis-ameliorating effect in *L. brevis* Bmb6.

## 1. Introduction

Inflammatory bowel disease (IBD) is a gastrointestinal tract disorder characterized by chronic inflammation of the mucosal cells. The incidence of IBD has been increasing rapidly since 1990, affecting one in 200–300 people in high-income countries. These epidemiological data suggest that a further increase in IBD incidence may pose a major health burden to the community [1]. Although the etiology of IBD is yet to be clarified, evidence suggests genetic elements, dietary patterns, and alteration of the gut microbiome as factors leading to immune-dysregulation and intestinal barrier dysfunction in IBD [2,3,4]. Interestingly, impaired tight junction (TJ) barrier function was observed in patients with IBD and an animal model, which significantly increased the risk of bacterial translocation and infusion of other harmful substances into the bloodstream, leading to bacteremia and organ failure [5,6,7]. Hence, maintaining healthy and functional TJ barrier integrity is of utmost importance to hinder the development of gastrointestinal and systemic disorders.

As vital components of the epithelial barrier, TJ proteins such as claudins, occludin, and zonula occludens (ZO) connect the adjacent epithelial cells and provide mechanical stability to facilitate intercellular communication and paracellular transport [8]. For instance, the absence of ZO-1 can delay the assembly of other TJ proteins in the epithelial layer, and the absence of claudin-1 can increase intestinal permeability, resulting in extensive loss of body electrolytes [9,10]. It has been reported that prolonged exposure to pro-inflammatory mediators and pathogenic bacteria and viruses results in the dysregulation of TJ barrier function, subsequently leading to intestinal epithelial damage and increased paracellular permeability, as observed in IBD [8,11,12]. Moreover, existing studies report that inflammation and TJ barrier dysfunction are closely associated, whereby either TJ barrier dysfunction leads to inflammation or vice versa. For instance, TJ disruption triggers the influx of immune cells and production of pro-inflammatory cytokines, thereby initiating the pro-inflammatory cascade, which in turn further deteriorates intestinal epithelial damage and tissue homeostasis [11,13].

As a member of the gut microbiota, *Lactobacillus* has been used as an indicator of healthy gut and for the treatment for various diseases, especially those related with gastrointestinal discomforts, such as antibiotic-associated dysbiosis, IBD, and lactose intolerance. Numerous studies have reported that treatment with *Lactobacillus* species ameliorates colitis by regulating host immune responses, primarily via suppressing the overexpression of inflammatory factors [14,15,16,17,18,19]. In our previous study, *L. brevis* Bmb6-containing fermented milk decreased the disease activity score (DAI) of dextran sulfate sodium (DSS)-induced colitis mice [20]. It was speculated that *L. brevis* Bmb6 reconstitute the integrity of the gut barrier by restoring the expression and localization of TJ proteins in the inflamed intestinal epidermal layer, thereby contributing to the colitis-ameliorating effect. In the present study, the regulatory effect of *Lactobacillus brevis* Bmb6 on the expression of inflammatory factors and TJ proteins was elucidated using a DSS-induced colitis mouse model. We found that *L. brevis* Bmb6 regulates the cross-talk between inflammatory mediators and TJ protein in DSS-induced colitis mice, which in turn restores gut epithelial structural integrity and relieves colitis symptoms.

## 2. Materials and Methods

### 2.1. Acid and Bile Acid Tolerance Assay

Lactic acid bacteria were isolated from the traditional homemade kimchi in Gwangju, Jeollanam-do, Korea, and maintained in de Man, Rogosa, and Shape (MRS) broth (Difco, Detroit, MI, USA). For acid tolerance assay, the viability of *Lactobacillus* strains grown in acidic MRS broth (pH 2.5 with 1000 unit/mL pepsin; Sigma-Aldrich, St. Louis, MO, USA) was determined after 2 h of incubation at 37 °C. For bile acid tolerance assay, the viability of *Lactobacillus* strains was determined by cultivating the strains in MRS broth supplemented with 0.3% ox-gall (*w*/*v*) for 48 h at 37 °C. In this experiment, MRS broth was used to resemble the nutrient-rich environment of the gastrointestinal tract and to prevent a false-negative result due to lack of nutrients in bacteria. *Lactobacillus* strains exhibiting strong tolerance to acidic and bile acid conditions were selected for subsequent analyses.

### 2.2. Preparation of Bacterial Cell Lysate

Activated *Lactobacillus* strains were centrifuged at 1500× *g* for 10 min at 4 °C, washed three times with phosphate-buffered saline (PBS), and resuspended in ice-cold PBS. The cell pellets were lysed using 0.1 mm glass beads (BioSpec Products Inc., Bartlesville, OK, USA) with the Micro-BeadBeater (BioSpec Products Inc.) for 3 min at 4400 rpm, with 15 s in an ice bath at the end of each minute. Unbroken cells and cell debris were removed via centrifugation at 12,000× *g* for 10 min at 4 °C, and supernatants were collected as cell lysates.

### 2.3. Determination of Antioxidant Activity

The radical scavenging potential of the cell lysate of selected *Lactobacillus* strains was determined via the 1-1-Diphenyl-2-picrylhydrazyl (DPPH) and superoxide dismutase (SOD) assays. For DPPH radical scavenging assay, 0.1 mL of the cell lysate from selected *Lactobacillus* strains were mixed with 1.4 mL of 0.1 mM DPPH solution, and 100 mg/mL ascorbic acid was used as the positive control. The change in absorbance was measured at 517 nm before and after incubation in the dark for 15 min at room temperature. The radical scavenging ability was calculated using Formula (1) [21].

(1) $$\mathrm{DPPH}\;\mathrm{radical}\;\mathrm{scavenging}\;\mathrm{activity}=\left[\frac{A_{517control}-A_{sample}}{A_{517control}}\right]\times 100\%,$$

The SOD assay was performed as previously described with some modifications [22]. First, 2.9 mL reaction mixture [13.3 mM methionine, 63.0 µM nitro-blue tetrazolium chloride, 0.1 mM EDTA, and 1.3 µM riboflavin in 50 mM phosphate buffer (pH 7.0)] was added to 0.1 mL of cell lysate from selected *Lactobacillus* strains. The reaction mixtures were exposed to an ultraviolet lamp at room temperature for 20 min. A non-irradiated complete reaction mixture was used as a blank. The absorbance was measured at 560nm, and SOD activity was calculated using Formula (2).

(2) $$\mathrm{SOD}\mathrm{activity}=\left(1-\frac{sample}{blank}\right)\times 100\%.$$

### 2.4. Determination of β-Galactosidase Activity

The β-galactosidase activity of cell lysates from selected *Lactobacillus* strains was determined according to the Miller’s method [23]. The reaction mixtures consisted of 2 mM *o*-nitrophenyl-β-d-galactoside (ONPG) in 0.05 M phosphate buffer (pH 7.0) and cell lysates of selected *Lactobacillus* strains. The reaction mixtures were incubated at 40 °C for 15 min and the reaction was stopped by adding 1.0 M Na_2_CO_3_. The yellowish end product of ONPG hydrolysis, *o*-nitrophenol, was collected by centrifugation at 12,000 rpm for 15 min at 4 °C and its absorbance was measured at 420 nm.

### 2.5. 16S rDNA Sequencing and Identification

Genomic DNA of the strain with prominent acid and bile tolerance, antioxidant activity, and B-galactosidase activity was extracted using PureHelix™ Genomic DNA Prep Kit (Nanohelix, Daejeon, Korea). The almost complete 16S rDNA region of the selected strain was determined by Macrogen (Seoul, Korea) using the primers 27F and 1482R for polymerase chain reaction (PCR) amplification and sequencing as detailed in the Macrogen service website (https://dna.macrogen.com/eng/support/ces/guide/ces_sample_prep.jsp). The 16S sequence was analyzed, and the 16S rDNA phylogenetic tree was constructed and visualized using NCBI (https://www.ncbi.nlm.nih.gov). The 16S rDNA sequence of *Enterococcus faecalis* V538 (AE0168830) was used as an outgroup.

### 2.6. Induction and Assessment of DSS-Induced Colitis

**Figure 1:**
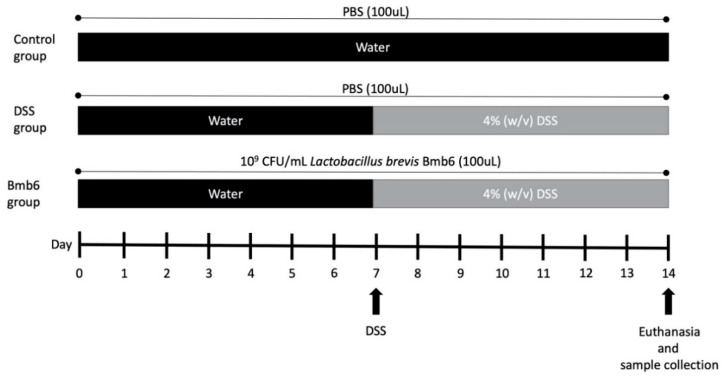
Experimental design to evaluate the colitis-ameliorating effect of *L. brevis* Bmb6 on dextran sulfate sodium- (DSS)-induced colitis mice. Eighteen mice were divided into three groups (*n* = 6); control, DSS, and Bmb6 groups. Bmb6 mice were administered with 10^9^ colony forming units (CFU)/mL, while control and DSS mice were administered with phosphate-buffered saline (PBS) by oral gavage throughout the experiment.

All animal experiments were approved and performed in accordance with the guidelines of the Institutional Animal Care and Use Committee of the Chonnam National University (CNU-IACUC-YB-2016-47). Eighteen five-week-old female C57BL/6J mice were purchased from Daehan Lab (Daejeon, Korea). The mice were housed and acclimatized for one week in the Animal Housing Unit under standard conditions of 22–25 °C, 50–60% humidity, and 12 h light/dark cycle. Standard mouse chow-diet and water were provided ad libitum. The mice were divided into three groups: control, DSS, and Bmb6 groups, with six mice in each group (*n* = 6). The experiment design is illustrated in Figure 1. In this experiment, PBS which had a similar osmolarity and ion concentration as the animal was used as an adjuvant. Mice in the control and DSS groups were orally administered with 100 µL PBS, while those in the Bmb6 group were orally administered with viable *L. brevis* BMB6 [10^9^ colony forming units (CFU) in 100 µL PBS] daily. At day 7, the drinking water in the DSS and Bmb6 groups was replaced with 4% (*w*/*v*) DSS in water until the end of the experiment. The disease activity index (DAI) was assessed daily based on a scoring system (Table 1) [24]. On day-14, the mice were sacrificed. The length of the colons was measured, and the colon contents were carefully collected.

### 2.7. Histological Assessment

The collected colon was washed three times with sterile PBS. A part of the colon tissue was then fixed with 10% phosphate-buffer formalin for 24 h. After fixation, the tissue sample was dehydrated through an ethanol series, followed by embedding in paraffin. The paraffin blocks were then sectioned (5 µm) and stained with hematoxylin-eosin for histological evaluation. For mucosal layer evaluation, Alcian blue was used to stain the mucin in the paraffin-embedded sections and the nuclei were counterstained with nuclear fast red. For immunofluorescence analysis, the cut sections were stained with ZO-1 (#61-7300; Invitrogen, Carlsbad, CA, USA) or claudin-1 antibodies (#71-7800; Invitrogen) and goat anti-rabbit Alexa Flour 488 secondary antibody (A-11008; Invitrogen). 4′,6-diamidino-2-phenylindole (DAPI; D-1306; Invitrogen) was used to counterstain cell nuclei. Slides were examined and analyzed using an epifluorescence microscope.

### 2.8. RNA Extraction and Gene Expression Analysis

Total RNA was extracted from the excised mouse colon using the RNeasy Mini Kit (Qiagen, Valencia, CA, USA) according to manufacturers’ protocol. Next, 2 μg of total RNA was used to synthesize complementary DNA (cDNA) using Maxime RT Premix Oligo (dT) RT-PCR kit (iNtRON Biotechnology, Inc., Seongnam, Korea). The primers used in the study are listed in Table 2. Glyceraldehyde-3-phosphate dehydrogenase (GAPDH) was used as an internal control. Quantitative-PCR was performed on the Bio-Rad thermal cycler (Bio-Rad Laboratory, Hercules, CA, USA). The PCR conditions were as follows: initial denaturation at 95 °C for 5 min, followed by 30 cycles of denaturation at 95 °C for 30 sec, annealing at 56 °C for 30 s, and extension at 72 °C for 5 min. The relative gene expression levels were determined by comparative analysis, using Formula (3).

(3) $$\mathrm{Relative}\;\mathrm{expression}\;=2^{-\left(\Delta Ct\right)},\;\mathrm{with}\;C_{t}=C_{t\;gene}-C_{t\;GAPDH}$$

### 2.9. Western Blotting

Total protein was extracted from the excised mouse colons using PRO-PREP protein extraction solution (iNtRON Biotechnology, Inc.). Briefly, 100 mg of the excised mouse colon tissue was immersed in 500 mL of PRO-PREP solution and homogenized using Q125 sonicator (QSonica, Sonicator, Newtown, CT, USA) for 5 min (pulsing mode of 15 s on and off) and 40% power in the ice bath. The mixture was then centrifuged at 13,000× *g* at 4 °C for 5 min, and the supernatant was collected as the extracted protein. The concentration of the extracted protein was determined using the Pierce™ BCA Protein Assay Kit (Thermo Fisher Scientific, Rockford, IL, USA). The extracted proteins (50 µg) were separated using 12% sodium dodecyl sulfate-polyacrylamide gel and electroblotted (Mini-PROTEAN^®^ II Cell Systems; Bio-Rad Laboratories, Hercules, CA, USA) onto the polyvinylidene difluoride membranes (PVDF; Bio-Rad Laboratories). The membranes were blocked with ZO-1 (#61-7800; Invitrogen), claudin-1 (#71-7800; Invitrogen), and β-actin (sc-4778; Santa Cruz Biotechnology, Inc., Dallas, TX, USA) antibodies in 5% (*w*/*v*) skim milk-supplemented with Tween 20-Tris-buffered saline (TTBS) overnight at 4 °C. After incubation, membranes were washed three times with TTBS and incubated with horseradish peroxidase-conjugated goat anti-mouse or anti-rabbit antibodies. Protein bands were then developed and detected with enhanced chemiluminescence, and the band density was determined using β-actin as the reference protein.

### 2.10. Statistical Analysis

All data are presented as the mean ± standard deviation from three independent runs or six animals per group (*n* = 3 or 6). Tukey’s multiple comparison test and repeat measure analysis of variance (ANOVA) were performed using SPSS 20 (SPSS, Inc., Chicago, IL, USA) with a *p* < 0.05 considered to be statistically significant.

## 3. Results

### 3.1. In Vitro Characteristic of L. brevis Bmb6

As shown in Table 3, the growth of all strains decreased under acidic environments. The number of viable cells was only reduced by one log_10_ in strains 2, 4, R10, P11, and Bmb6, whereas strains 1 and 5 were unable to survive in the acidic environment. For bile acid tolerance assay, strain Bmb6 exhibited prominent tolerance to bile acid, with a growth of two log_10_ CFU/mL in the presence of 0.3% (*w*/*v*) ox-gall.

### 3.2. Antioxidant Activity

**Figure 2:**
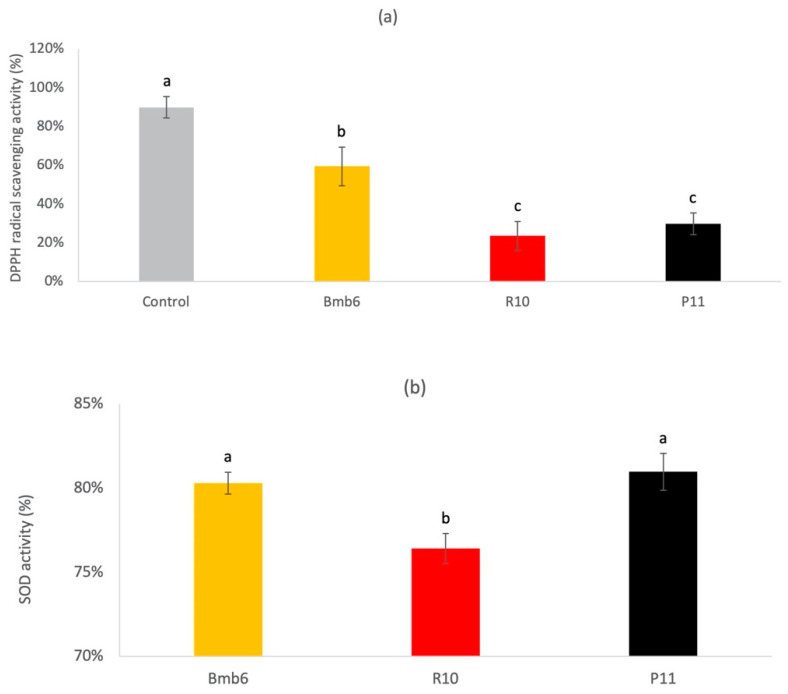
(**a**) 1-1-Diphenyl-2-picrylhydrazyl (DPPH) radical scavenging activity, and (**b**) superoxide dismutase (SOD) activity of different lactobacilli. Data represent the mean ± standard deviation from three independent experiments (*n* = 3). Tukey’s multiple comparison test was performed, and different lowercase letters indicate statistically significant differences (*p* < 0.05).

The DPPH radical scavenging assay results (Figure 2a) demonstrated that *Lactobacillus* strain Bmb6 was the most effective DPPH radical scavenger (59.48%), as compared to strains R10 (23.59%) and P11 (29.85%). The SOD assay results (Figure 2b) showed that both Bmb6 and P11 exhibited similar enzymatic activities of 80.28 and 80.97%, respectively, which were significantly higher than those of R10 (76.41%). Hence, our data suggested that Bmb6 strain exhibited a more significant antioxidant activity than R10 and P11 strains.

### 3.3. β-Galactosidase Activity

**Figure 3:**
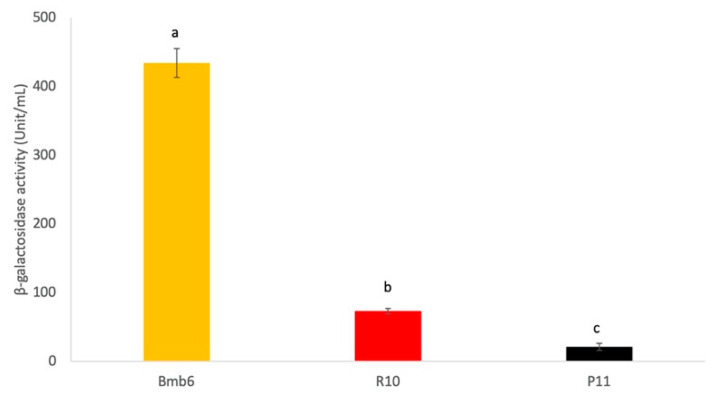
β-galactosidase activity of different lactobacilli. Data represent the mean ± standard deviation from three independent experiments (*n* = 3). Tukey’s multiple comparison test was performed, and different lowercase letters indicate statistically significant differences (*p* < 0.05).

The β-galactosidase activity was determined by measuring the production of yellowish o-nitrophenyl, the end product of ONPG hydrolysis (Figure 3). Based on our results, strain Bmb6 showed the highest (434 ± 21 unit/mL) while strain P11 showed the lowest β-galactosidase activity (21 ± 5 unit/mL), revealing the probiotic potential of strain Bmb6.

### 3.4. 16S Identification

16S rDNA analysis using BLAST showed that strain Bmb6 had 100% homology with *L. brevis*. Moreover, a neighbor-joining tree (data not shown) was constructed based on the 16S rDNA, and a close grouping of Bmb6 with the other eighteen *L. brevis* strains was observed, which was in agreement with the BLAST results, thus validating its identity as L. brevis Bmb6.

### 3.5. Effects of L. brevis Bmb6 on DSS-Induced Colitis Mice

**Figure 4:**
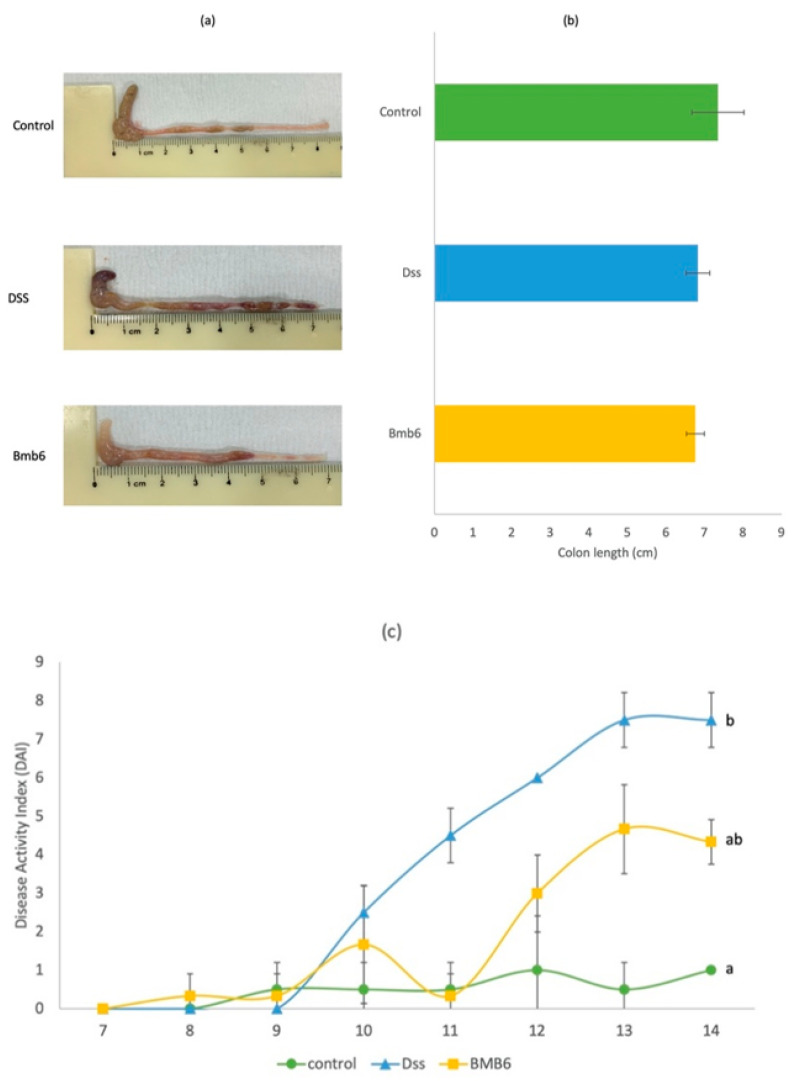
Effects of *L. brevis* Bmb6 on the (**a**,**b**) colon length and (**c**) disease activity (DAI) score of DSS-induced colitis mice. Data represent the mean ± standard deviation of six mice from each treatment group (*n* = 6). Repeated measure ANOVA was performed for DAI score, and different lowercase letters indicate statistically significant differences (*p* < 0.05).

The control mice exhibited the longest colon length (7.35 ± 0.68 cm), with normal shaped feces in the light red color colon (Figure 4a,b). In comparison, DSS mice had a shorter colon, with an average length of 6.83 ± 0.31 cm and dark red color, with intestinal hemorrhage. Meanwhile, the colons of Bmb6 mice had an average length of 6.77 ± 0.23 cm. There was no significant difference in the colon length among the three groups of mice (control, DSS, and Bmb6). However, the colons of the Bmb6 group had a lighter color than those of the DSS group, indicating that *L. brevis* Bmb6-treated mice display higher alleviation of intestinal hemorrhage than DSS-induced mice.

On day-10, the DAI score began to rise in the DSS group, and loose, unformed, and bloody stools were visible to the naked eye (Figure 4c). The DAI score of the DSS group further increased to 7.50 ± 0.71 on day 13, following which mice displayed a drastic decrease in body weight and watery diarrhea with blood till the end of day 14. Similar to the DSS group, the DAI score of the Bmb6 group started to increase on day 10. Administration of *L. brevis* Bmb6 significantly alleviated colitis symptoms, as loose and hemoccult feces and a lower DAI score of 4.33 ± 0.58 was observed at the end of the study, as compared to the DSS group. Meanwhile, the control group showed no colitis symptoms, and the DAI score fluctuated around 1.0.

### 3.6. Histological Analysis of Colon Sections

**Figure 5:**
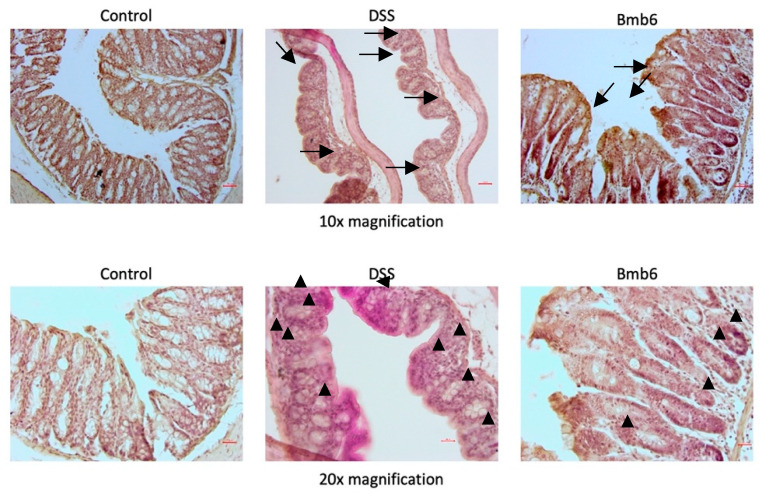
Hematoxylin-eosin staining of colon sections in DSS-induced colitis mice. The DSS group showed an intensive loss of intact epithelial structure (arrow) and infiltration of immune cells (arrowhead); the Bmb6 group showed slight epithelial structural damage (arrow) and infiltration of immune cells (arrowhead). Scale bar = 50 μm for 10× magnification; scale bar = 25 μm for 20× magnification.

Hematoxylin-eosin-stained colon sections showed normal and intact intestinal epithelial structures with no symptoms of immune cell infiltration in the control group (Figure 5). However, intense infiltration of immune cells and severe epithelial structural damage was observed in the colon of DSS-induced mice. In contrast, the colon of *L. brevis* Bmb6-treated mice was greatly improved, with only slight epithelial structural damage and immune cell infiltration.

**Figure 6:**
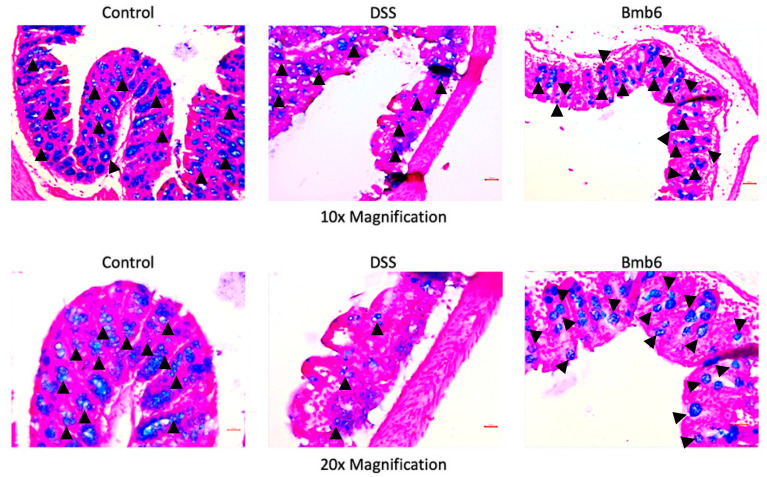
Alcian blue staining of colon sections in DSS-induced colitis mice. Mucin-secreting goblet cells (arrowhead) were abundantly distributed in the control group. The number of mucin-secreting goblet cells was greatly reduced, with a limited number of goblet cells (arrowhead) scattered around the colon in the DSS group. Treatment with *L. brevis* Bmb6 protected against DSS-induced damage by increasing the number of mucin-secreting goblets (arrowhead) in the colon. Scale bar = 50 μm for 10× magnification; Scale bar = 25 μm for 20× magnification.

The Alcian blue-stained colon micrographs (Figure 6) showed that the mucous layer and goblet cells were well protected and preserved in the control group. However, the mucous layer was disrupted, and only a limited number of goblets cells were detected in the DSS group. In contrast, a higher number of goblet cells and only a slight alteration in mucus integrity was observed in the Bmb6 group.

### 3.7. Effects of L. brevis Bmb6 on TJ Proteins

**Figure 7:**
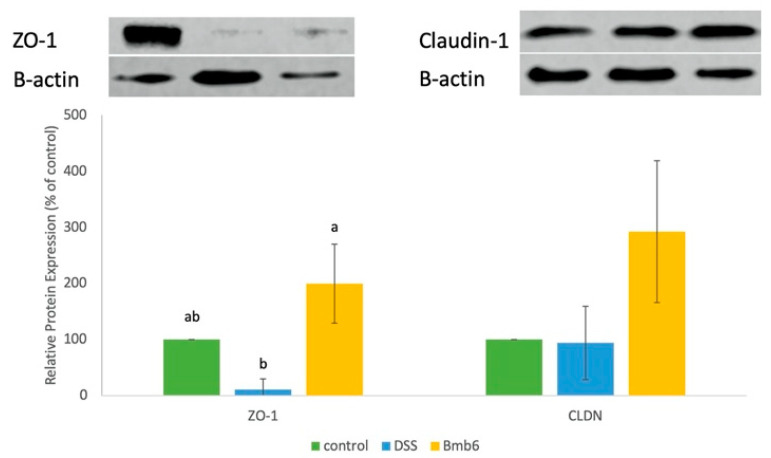
Western blot analysis of the expression ZO-1 and claudin-1 in DSS-induced colitis mice. Data represent the mean ± standard deviation (*n* = 6 mice per group). Tukey’s multiple comparison test was performed, and different lowercase letters indicate statistically significant differences (*p* < 0.05).

As shown in Figure S1a, ZO-1 protein was detected in the colon sections of the Bmb6 group but was absent in the DSS group. Moreover, western blot analysis showed that the expression of ZO-1 was significantly increased by 192.12 ± 70.44% in the Bmb6 group, but significantly decreased by 89.02 ± 18.92% in the DSS group (Figure 7). Meanwhile, claudin-1 was detected in the colon of control, DSS, and Bmb6 groups (Figure S1b). As revealed by western blot analysis, a non-significant increase in the expression of claudin-1 was observed in the Bmb6 group, compared to the DSS group (Figure 7). The overall findings indicate the *L. brevis* Bmb6 ameliorates colitis by partially recovering the expression of ZO-1 protein.

### 3.8. Effects of L. brevis on Colonic Inflammatory Cytokine Expression

**Figure 8:**
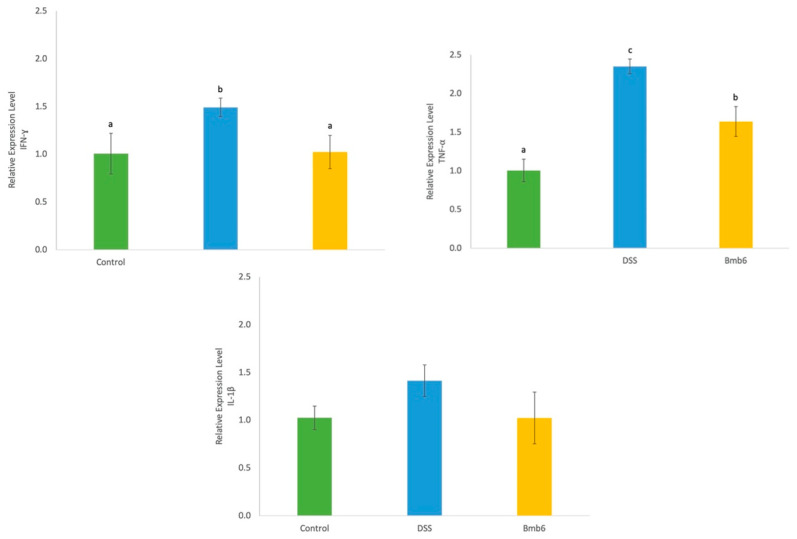
Relative gene expression of inflammatory cytokines (IFN-γ, TNF-α, and IL-1β) in the colon of DSS-induced colitis mice. Data represent the mean ± standard deviation (*n* = 6 mice per group). Tukey’s multiple comparison test was performed, and different lowercase letters indicate statistically significant differences (*p* < 0.05).

As shown in Figure 8, the gene expression of interferon (IFN)-γ and tumor necrosis factor (TNF)-α was significantly increased upon DSS treatment. However, treatment with *L. brevis* Bmb6 suppressed IFN-γ expression to a level similar to that in control. Moreover, TNF-α gene expression was significantly suppressed in the Bmb6 group compared to that in the DSS group but remained at a higher level than the control group. Meanwhile, no difference in interleukin (IL)-1β gene expression was observed between the control, DSS, and Bmb6 groups.

## 4. Discussion

Numerous studies have reported that consumption of fermented food helps in alleviating gastrointestinal disorder symptoms [28,29,30,31,32]. Notably, the functional microorganisms, especially those from the genera *Bifidobacterium*, *Lactobacillus*, *Lactococcus*, and *Pediococcus*, and their bioactive metabolites found in fermented food, have shown to having been responsible for these functions [19,20,28,30,31,32,33,34,35]. Hence, in this study, potential lactic acid bacteria were isolated from the local homemade fermented food, kimchi, to investigate their regulatory effects on alleviating colitis symptoms, especially on TJ recovery. Our preliminary studies indicated that *L. brevis* Bmb6 possess several probiotic traits, including tolerance to acidic and bile acid conditions, which resemble the gut environment, and a prominent β-galactosidase activity.

In addition, *L. brevis* Bmb6 possesses strong antioxidant potential, as indicated by its prominent radical scavenging activity in the preliminary DPPH scavenging and SOD assays. A growing body of evidence suggests that elevated reactive oxygen and nitrogen species are closely associated with intestinal inflammation [36]. Accumulation of these radicals creates a high-oxidative stress environment, damaging the mucin layer and epithelial cells, subsequently stimulates the infiltration of immune cells and secretion of inflammatory mediators, and initiates gut inflammation [37,38]. Several studies have shown that treatment with SOD or high-SOD-producing *Lactobacillus* significantly reduces colonic myeloperoxidase level, oxidative stress, and inflammation in DSS-induced colitis mice [39,40,41,42]. Although the prominent in vitro radical scavenging activities of *L. brevis* Bmb6 suggest its potential role in alleviating colitis, its effect on lowering oxidative stress in vivo has not yet been evaluated.

For in vivo evaluation, DSS was used to induce colitis in mice. The clinical symptoms of DSS-induced mice are similar to those of patients with IBD, which include bloody stool, diarrhea, elevated inflammatory biomarkers, and drastic weight loss [43]. In our previous study, we reported that *L. brevis* Bmb6-containing fermented milk significantly improved the DAI score in DSS-induced mice [20]. This is in agreement with our current results which showed that the DAI score was significantly decreased in the Bmb6 group, with no drastic weight loss, and only non-forming hemoccult stools were observed. Despite weight loss and bloody diarrhea, shortened colon length, colon hemorrhage, and destruction of intestinal epithelial structures are typically observed in DSS-induced colitis mice [44,45,46]. In this study, administration of *L. brevis* Bmb6 alleviated colon hemorrhage while preserving the intact intestinal epithelial structure but showed no effect on restoring the colon length. Histological analyses revealed that *L. brevis* Bmb6 treatment alleviated colonic hemorrhage in DSS-induced colitis mice by preserving the integrity of the epithelial structure and preventing mass infiltration of immune cells and destructive damage of goblet cells in the colon, thereby contributing to the lower DAI score and absence of bloody diarrhea in the Bmb6 group. Preservation of goblet cells is crucial for maintaining mucosal barrier integrity via the secretion of mucins and trefoil factors [47]. Therefore, our findings suggest that the colitis-ameliorating effect of *L. brevis* Bmb6 was partly attributed to its ability to preserve the integrity of the intestinal epithelial structure.

The expression and localization of TJ proteins, such as ZOs, occludin, and claudins play a key role in regulating intestinal barrier functions. Impairment of the TJ barrier increases the paracellular permeation of pro-inflammatory molecules and activates mucosal immune response, resulting in chronic inflammation and tissue damage [6,48,49,50]. In the current study, the expression and localization of ZO proteins, particularly ZO-1, were investigated. ZO proteins have been known for regulating the assembly and maintenance of TJ structure. For instance, the most well-studied ZO protein, ZO-1, was shown to be involved in the early assembly of TJ proteins into the cells and connecting adjacent cells in both cell cultures and animal models. Moreover, the absence of ZO-1 protein resulted in impaired TJ function, wherein TJ formation was delayed, and no occludin and claudins were detected [10,51]. Consistent with previous studies, the expression of ZO-1 was reduced in the DSS group. However, the administration of *L. brevis* Bmb6 significantly improved the expression of ZO-1 in the Bmb6 group, indicated by western blot analysis. Hence, our findings indicate a strong correlation between ZO-1, DAI score, and colon tissue histology, highlighting the role of *L. brevis* Bmb6 in TJ recovery and preservation of the intestinal epithelial structure, thereby ameliorating colitis.

In addition to ZO-1, the expression of claudins, which are critical components of the TJ barrier, was also investigated in the present study. Claudins connect adjacent cells via heterophilic and hemophilic interactions, forming pores and barriers for paracellular permeation of specific molecules in various tissues [52,53,54]. For instance, the critical role of claudin-1 in TJ barrier function was demonstrated using claudin-1 knockout mice, who displayed impaired epidermal barrier function and died within 24 h owning to excessive loss of body electrolytes [9,55]. However, the role of claudin-1 in IBD remains unclear. A number of studies have reported an elevation in claudin-1 expression in both experimental colitis models and patients with IBD [56,57,58]. Meanwhile, studies have also reported a decrease in claudin-1 expression in an experimental colitis model [59,60,61]. Notably, our findings showed that the expression of claudin-1 was not significantly different among control, DSS, and Bmb6 groups. We were unable to correlate the results of claudin-1 expression with the DAI score and histological analyses data. Hence, we were unable to conclude the role of claudin-1 in the experimental colitis model. Further studies are needed to clarify the role of claudin-1 in the pathogenesis of colitis.

Abnormal and uncontrolled secretion of inflammatory cytokines is also commonly seen in patients with IBD. Among the vast number of cytokines, TNF-α plays a key role in the pathogenesis of IBD by inducing inflammation and cell apoptosis, and subsequently intestinal TJ barrier defect in intestinal epithelial cells [62,63]. As a pro-inflammatory cytokine, TNF-α activates NF-**κ**B and initiates the pro-inflammatory cascade by recruiting other pro-inflammatory factors, such as IFN-γ and IL-1β, further intensifying inflammatory reaction and intestinal dysfunction [48,64]. Therefore, elevated levels of IFN-γ and IL-1β are commonly detected in patients with IBD. An increase in IFN-γ and IL-1β levels has been shown to alter the expression and distribution of TJ proteins among the intestinal epithelial cells, resulting in hyper-paracellular permeation [65,66,67]. Hence, in this study, the gene expression of the pro-inflammatory cytokines TNF-α, IFN-γ, and IL-1β was assessed. Treatment with *L. brevis* Bmb6 significantly suppressed the gene expression of TNF-α and IFN-γ, but not IL-1β in DSS-induced colitis mice. These outcomes were consistent with the histological analyses data showing improved protein expression of the TJ protein, ZO-1, and well-preserved intestinal epithelial structure in *L. brevis* Bmb6-treated DSS-induced colitis mice, thereby indicating that regulation of inflammation conditions can restore the expression of TJ protein or vice versa.

Taken together, *L. brevis* Bmb6 isolated from the fermented food, kimchi, exerted significant colitis-ameliorating effects through suppression of pro-inflammatory cytokines, improvement of ZO-1 protein expression, and preservation of the intestinal epithelial structural integrity. Our findings showed the colitis-ameliorating effects in *L. brevis* Bmb6 could be attributed to a close association between inflammation and TJ barrier functions, wherein a reduction in inflammatory response can restore the expression of TJ protein, ZO-1, and vice versa. Notably, the expression of claudin-1 remained controversial in the present experimental colitis model. Further investigation is needed to clarify the role of claudin-1 in IBD pathogenesis.

## Figures and Tables

**Table 1:** Scoring system for DAI ^1^.

Score	Weight Loss (%)	Stool Consistency ^2^	Gross Bleeding
0	0	Normal	Negative
1	1–5	Loose	Negative
2	6–10	Loose	Hemoccult positive
3	11–15	Diarrhea	Hemoccult positive
4	>15	Diarrhea	Bleeding

^1^ Disease activity index, DAI=(score of weight loss+stool consistency+gross bleeding)÷3. ^2^ Normal = well-formed pellets; Loose = Pasty stool that does not stick to the anus; diarrhea = liquid stool that sticks to the anus.

**Table 2:** Oligonucleotide primers used for quantitative polymerase chain reaction (PCR) analysis.

Genes	Oligonucleotide Sequences	References
*IL-1* *β*	Forward: TTGACGGACCCCAAAAGATG	[25]
	Reverse:AGAAGGTGCTCATGTCCTCA	
*TNF-* *α*	Forward: TCTCATCAGTTCTATGGCCC	[26]
	Reverse: GGGAGTAGACAAGGTACAAC	
*IFN-* *γ*	Forward: CTGAGACAATGAACGCTACACACTGC	[27]
	Reverse: AACAGCTGGTGGACCACTCGGAT	
*GAPDH*	Forward: CAAAAGGGTCATCATCTCTG	[25]
	Reverse: CCTGCTTCACCACCTTCTTG	

The relative gene expression was determined by quantitative PCR analysis with the PCR conditions of initial denaturation at 95 °C for 5 min, followed by 30 cycles of denaturation at 95 °C for 30 s, annealing at 56 °C for 30 s, and extension at 72 °C for 5 min.

**Table 3:** Tolerance of *Lactobacillus* strains to acidic and bile acid conditions.

*Lactobacillus* Strains	Acid Tolerance ^1^ (log_10_ CFU/mL)			Bile Acid Tolerance ^2^ (log_10_ CFU/mL)		
	0 h	1 h	2 h	0 h	24 h	48 h
1	5.71 ± 0.00	5.66 ± 0.00	0.17 ± 0.29 ^c^	5.61 ± 0.01	6.79 ± 0.00	6.82 ± 0.00 ^b,c^
2	5.72 ± 0.00	5.72 ± 0.04	4.67 ± 0.00 ^a^	5.76 ± 0.00	6.87 ± 0.00	5.72 ± 0.00 ^f^
3	5.83 ± 0.02	4.63 ± 0.00	0.13 ± 0.23 ^c^	4.84 ± 0.00	6.88 ± 0.00	5.54 ± 0.05 ^e^
4	5.74 ± 0.01	5.78 ± 0.01	4.67 ± 0.00 ^a^	4.80 ± 0.00	5.66 ± 0.00	5.52 ± 0.00 ^e^
5	6.17 ± 0.01	5.63 ± 0.05	0.14 ± 0.25 ^d^	5.73 ± 0.00	6.83 ± 0.00	6.72 ± 0.00 ^d^
8	6.76 ± 0.05	6.73 ± 0.00	4.58 ± 0.01 ^b^	5.70 ± 0.00	6.87 ± 0.00	6.78 ± 0.03 ^b^
9	6.84 ± 0.05	5.68 ± 0.00	4.53 ± 0.00 ^b^	5.84 ± 0.00	6.92 ± 0.00	6.92 ± 0.00 ^b^
R10	6.81 ± 0.00	6.74 ± 0.00	5.52 ± 0.01 ^a^	5.84 ± 0.00	6.92 ± 0.04	6.93 ± 0.00 ^b^
P11	6.81 ± 0.01	6.82 ± 0.00	5.75 ± 0.01 ^a^	5.85 ± 0.00	6.85 ± 0.04	6.85 ± 0.01 ^b^
Bmb6	6.86 ± 0.00	5.71 ± 0.00	5.76 ± 0.01 ^a^	5.83 ± 0.00	6.90 ± 0.00	7.94 ± 0.00 ^a^

Data represent the mean ± standard deviation from three independent experiments (*n* = 3). Tukey’s multiple comparison test was performed, and different lowercase letters (a, b, c, d, e, and f) indicate a significant difference between the change in the number of viable *Lactobacillus*. ^1^ Acid tolerance: *Lactobacillus* strains grown in acidic MRS medium (pH 2.0) with 1000 unit/mL pepsin at 37 °C for 2 h; ^2^ Bile tolerance: *Lactobacillus* strains growth in de Man, Rogosa and Shape (MRS) medium supplemented with 0.3% (*w*/*v*) ox-gall at 37 °C for 48 h.

## Supplementary Materials

The following are available online at https://www.mdpi.com/2304-8158/9/7/864/s1, Figure S1: Effects of *L. brevis* Bmb6 on the localization of TJ proteins (**a**) ZO-1 and (**b**) claudin-1, in the colon of DSS-induced colitis mice.
