# Supplementary material for: Regulatory Effect of Lactobacillus brevis Bmb6 on Gut Barrier Functions in Experimental Colitis
Source: Foods. 2020 Jul 2;9(7):864. doi: 10.3390/foods9070864 (PMC7404641; doi:10.3390/foods9070864)
Supplement: Supplementary file 1 [file foods-09-00864-s001.pdf]

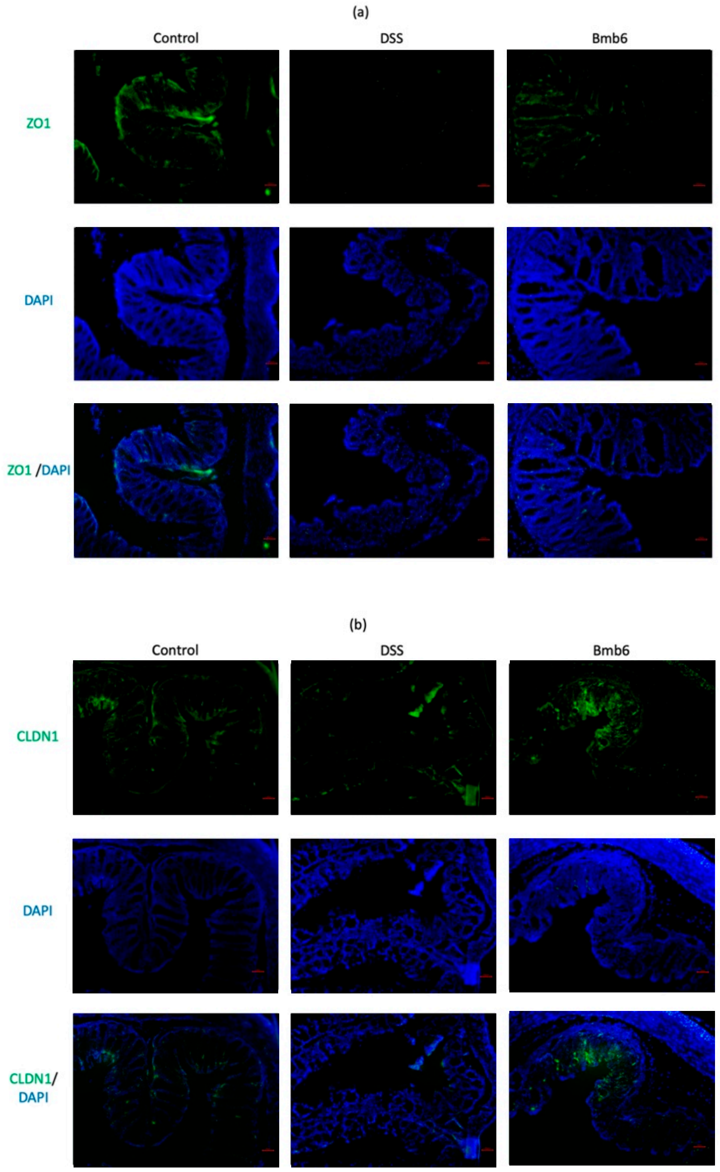

Figure S1. Effects of *L. brevis* Bmb6 on the localization of TJ proteins **(a)** ZO-1 and **(b)** claudin-1, in the colon of DSS-induced colitis mice. Cut sections were blocked with primary antibodies, followed by Alexa Fluor 488 secondary antibody. Green color represents TJ proteins, and blue represents the nuclei; scale bar represent 50 µm.
